# The Influence of Age, Gender and Religion on Willingness to be an Organ Donor: Experience of Religious Muslims Living in Sweden

**DOI:** 10.1007/s10943-018-0670-7

**Published:** 2018-07-13

**Authors:** Ferid Krupic, Olof Westin, Mårten Hagelberg, Olof Sköldenberg, Kristian Samuelsson

**Affiliations:** 1 Department of Orthopaedics, Institute of Clinical Sciences, Sahlgrenska Academy, University of Gothenburg, Göteborgsvägen 31, 431 80 Mölndal, Sweden; 2 Department of Clinical Sciences, Danderyd Hospital, Karolinska Institute, Stockholm, Sweden

**Keywords:** Organ donation, Knowledge, Information, Age, Gender, Religion, Qualitative research

## Abstract

The transplantation of organs is one of the most successful medical advances in recent decades, and transplantation is the treatment of choice for severe organ failure worldwide. Despite this situation and the general acknowledgment of organ donation (OD) as a global priority, the demand for organs outstrips the supply in virtually every country in the world. The study aims to elucidate whether age, gender and religion influence decision-making about organ donation in religious Muslims living in Sweden Data were collected through three group interviews using open-ended questions and qualitative content analysis. Twenty-seven participants, 15 males and 12 females from four countries, participated in the focus group interviews. The analysis of the collected data resulted in three main categories: “Information and knowledge about organ donation,” “The priorities when deciding about organ donation” and “The religious aspects of organ donation,” including a number of subcategories. Good information about and knowledge of OD, priorities in OD, importance of the fact that religion must be studied and taught daily and religious education were only a few of the factors informants emphasized as predictors of the total and successful donation of organs. Age, gender or religion did not have an impact on organ donation. High levels of education through religious education and good information via various media, as well as a good knowledge of the Swedish language, are predictors of improved OD. In order to overcome religious ideology as a source of misinformation relating to OD and to promote increased OD in the future, specific intervention studies and the improved involvement of religious communities and education in schools and the healthcare system are vital and must be a starting point for improved OD.

## Introduction

The immigrant policy in Sweden was founded in 1950 when there were about 3% of the population in Sweden or about 1500 inhabitants born in other countries but living in Sweden. Since then, immigration policy has not changed and there has been a steady inflow of migrants from around 10–15,000 every year. Since 1990, the influx of refugees has increased and 1992 about 100,000 migrants arrived in Sweden, and in 2016 approx. 180,000 immigrants. The immigrants are mostly Muslims, different ages and gender and of various professional disciplines (The Swedish Health and Medical Act 2014). The views held by influential religious people are important, because these individuals often guide public attitudes and behavior. The incidence of organ failure has been increasing all over the world, with many patients dying while waiting for donors. The final result relating to organ donation (OD) depends on the final decision of people directly linked to the healthcare system (Council of Europe 2012). Current Swedish legislation states that, when all brain functions are totally and irrevocably lost, a person may be declared dead (Wilow 2013). This means that integral organs and tissues can be utilized for transplantation surgery, because breathing and circulation can be maintained artificially. In Sweden every year, 250–300 people die in a way that makes them potential organ donors. Of these, 100–150 are medically suitable and of an age appropriate to become donors (The Swedish Health and Medical Act 2014). Healthcare professionals have a very important role to play in providing support, in the form of information, to transplant patients and to those who choose to donate organs. Healthcare professionals should also ensure that dying individuals and their families have understood the information given in order to make an informed decision on whether or not they wish to donate (Wang et al. 2006). However, the demand for organs is greater than the supply, despite well-functioning laws and competent healthcare professionals throughout the world (Park et al. 1999; Council of Europe 2012). Previous studies have shown that there are complex factors that influence public attitudes to deceased OD. They include race, ethnicity, gender, (Chen et al. 2006) and the age of the participants, (Miles and Frauman 1998; Conesa et al. 2003) where those who have higher levels of educational attainment and income on average (Conesa et al. 2003, 2006; Rumsey et al. 2003) are more likely to have positive attitudes to deceased OD (Rumsey et al. 2003). A greater knowledge of OD also predicts positive attitudes to OD (Wakefield et al. 2010). Additionally, psychosocial characteristics appear to be associated with OD attitudes, as individuals with altruistic tendencies are more likely to find deceased OD favorable (Wakefield et al. 2010). Previous studies even show that the high need and low supply among minority ethnic populations present problems in achieving the optimal match of blood group and tissue type, where these are less common among the majority population, resulting in patients from minority ethnic groups spending a longer time on the transplantation waiting list (Siminoff et al. 2006; Morgan et al. 2006). Research investigating the lower donation rates among ethnic minorities indicates that, although minority ethnic groups generally support the “gift” of life, they are often less well informed about OD and more commonly hold beliefs that are identified as barriers to deceased OD (Davis and Randahawa 2004; Kurtz et al. 2007; Krupic et al. 2017). All religions in the world traditionally believe that saving life is more important than any other objections that may arise and all the main religions approve of OD (Bruzzone 2008). Islam allows organ and tissue transplantation in order to save human life or vital organs (Duzenly 2005). Despite the fact that the majority of Islamic religious officials believe OD to be desirable in Islamic culture, only half of them would donate organs after their death and only one in three would consent to the use of organs for their relatives after death. Religious leaders are frequently consulted for advice on OD; more than half of those who responded in one study reported being asked for input, as family members, regarding organ donation (Sharif 2012).

### Aim of the Study

The aim of the qualitative study was to elucidate whether age, gender and religion influence decision-making about OD in religious Muslims living in Sweden.

## Methods

### Design

The study was designed as a qualitative study using data from interviews with participants from Bosnia and Herzegovina, Macedonia, Kosovo and Croatia. The data were collected through three focus group interviews (McLafferty 2004), with 8, 9 and 10 participants in each group.

### Participants

The inclusion criteria were participants from Bosnia and Herzegovina, Macedonia, Kosovo and Croatia who were more than 20 years old, who had lived in Sweden for more than 10 years and who described themselves as religious Muslims. Twenty-seven participants were invited to participate in the study and they all agreed. There were 12 women and 15 men, aged 21–75 years (mean 47.6 years). The men were aged 31–74 (mean 56.5 years) and the women 21–60 (mean 39.8 years). The participants were assigned to three age groups. In the first, there were eight participants, aged 20–39 years (three women and five men). In the second, there were ten participants, aged 40–59 years (four women and six men), and, in the third group, there were nine participants, aged 60–75 years (five women and four men). The interviews and all the communication were carried out in the Bosnian and Swedish languages. The demographic and clinical characteristics of the informants are shown in Table 1.

**Table 1:** Characteristics of the study population

Variables	Numbers
Gender	
Male	15
Female	12
Total	27
Educational level	
Elementary school	1
High school/university	18
Master’s	4
Doctoral/postdoctoral education	6
Total	27
*Qur’an course education*	*27*
Age (years)	
20–30	6
30–39	2
40–50	5
51–59	5
60–70	7
> 70	2
Total	27
Employment	
Employed	22
Unemployed	1
Pensioner	4
Total	27

### Procedure

Data were collected through group interviews by the first author, using individualized open-ended questions, following an interview guide inspired by Kvale (1997). The interviews were performed from May 2016 to November 2016. They began with small talk. The opening questions were “What do you know about organ donation?” and “Would you consider donating your own organs or organs from a member of your family?”. The initial questions were supplemented with other short questions, such as “Could you please tell me more about that?” and “What do you mean by that?”. All contact with the participants was arranged in collaboration with a key person in a Bosnia and Herzegovina and Croatia association in the western part of Sweden. Participants who fulfilled the inclusion criteria were asked to participate in the study. When the key person had recruited enough participants, the first author of the study was contacted and the interview was arranged. Printed information about the aim and background of the study was distributed to the participants and repeated to them orally before the interview. The interviews were carried out in groups of eight to ten participants and held in the facilities of the Bosnian and Croatian association and the Bosnian youth association. The participants were encouraged to speak freely using their own words and the interviewer encouraged the participants to respond to the questions as comprehensively as possible. The interviews were carried out in Bosnian and Swedish by the first author, who is bilingual. There were some younger participants who chose to speak Swedish. All the interviews were therefore translated first into Swedish by the author and a professional translator checked the translation. The interviewer only interrupted to ask questions or to follow-up on the information given. All the participants gave their signed informed consent before the interviews. The interviews lasted between 75 and 125 min and were taped and transcribed verbatim.

### Data Analysis

The qualitative content analysis method, in accordance with Graneheim and Lundman (2004), was chosen for the analysis and interpretation of the collected data. This method is suitable for the analysis of qualitative data because, by using this method, the researcher is able to condense a large amount of data into a small number of codes, subcategories, categories and themes. We conducted a manifest analysis of the text. The transcripts were read carefully in order to identify the informants’ experiences and conceptions. The analysis then proceeded by extracting meaningful units, consisting of one or several words, sentences, or paragraphs, containing aspects related to each other and addressing a specific topic in the material. Meaningful units, related to each other through their content and context, were then abstracted and grouped together into a condensed meaningful unit, with a description close to the original text. The condensed text was further abstracted and labeled with a code. Thereafter, codes that addressed similar issues were grouped together, resulting in subcategories. Subcategories that focused on the same problem were brought together, in order to create more extensive conceptions, which addressed an obvious issue (Graneheim and Lundman 2004). The results are presented with direct quotations from the interviews.

### Ethics

Since there was no physical intervention and no information on individual health issues was involved in the study, there was no need to involve the ethical board, according to Swedish law (2015). The World Medical Association Declaration of Helsinki (1964) was followed carefully. The informants’ identities were protected; that is, their names and personal identity numbers were not stated in the recordings or any publications. The audiotapes used for the interviews were stored in a locked safe at the hospital. The identity of the participants could therefore not be traced. The study information given to the participants included its voluntary nature and the fact that they could withdraw at any time without incurring penalties or losing access to services.

## Results

The analysis of the text resulted in three main categories and six subcategories, based on how the participants described their situation regarding OD in Sweden. The categories, together with the subcategories, are presented in Table 2. The categories were information and knowledge about OD, the priorities when deciding about OD and the religious aspects of OD.

**Table 2:** Overview of the theme, categories and subcategories

Categories	Subcategories	Theme
Information and knowledge about organ donation	Obtaining information through the media Obtaining information from medical institutions Having a donor card	
The priorities when deciding about organ donation	Donation of organs to family members Donation of organs to friends Organ donation to acquaintances and others	Age, gender and religion as predictors of organ donation
The religious aspects of organ donation	The attitude of religion to donating organs Religious education	

### Information and Knowledge About Organ Donation

In the first main area, all the participants in the study noted the importance of receiving information and having a knowledge of OD. They also noted that the key to the donation of organs lies in the amount of knowledge and level of information about OD. The participants in the study showed in their discussions that they were well informed and willing and that they had knowledge of OD. They described a variety of sources for acquiring knowledge and obtaining information. The informants even stated that, just as society is obliged to inform people, people have a duty to participate personally in various social projects, such as OD. As the last link in the chain of the decision to donate organs, the respondents mentioned the importance of having donor cards.

#### Obtaining Information Through Various Forms of Media

All the participants in this study indicated that one of the main ways of acquiring knowledge and information about OD is through the media. Most of them watched Swedish television and listened to Swedish radio and regularly used the Internet. The participants mentioned that it is important to accept all the customs and values of the country in which one lives and not just live in the past. Furthermore, the informants mentioned that information on OD is all around us, people just need to listen and take advantage of it.

One respondent said:

All the information is available on radio and television… and there is the internet.

Another respondent noted:

Everything you need can be found on the internet… Google is the best.

#### Obtaining Information from Medical Institutions

The importance of information on OD provided by healthcare institutions was a conclusion reached by the respondents in the study. The informants noted that it was mostly a pleasure to receive information from these institutions. They also stated that they are the most important and competent institutions when it comes to providing information on OD, as organ transplantations are performed at medical institutions. The participants emphasized that the Swedish healthcare organizations inform people effectively about OD and the participants were satisfied with that information.

One respondent said the following:

There is a section on organ transplants, there are persons who have previously donated organs, there is no information at the hospital and there is the number 1177 or www.1177.se where we can obtain information about everything that interests us.

Another also added:

Yes… everywhere and every time you contact them they are so kind that you almost have a death wish so you can contact them again.

#### Having a Donor Card

The participants in the study agreed on the importance of having a donor card. All of them have a donor card and some of them have a card both from Sweden and from the country where they were born. The participants were willing to donate their organs and each donor card was marked “Yes.” It was also pointed out that the ownership of donor cards is very important in urgent medical cases, where it is a matter of life or death and where minutes can save another life if the person who has died is a potential organ donor.

One woman described it this way:

There are many cases where a person dies and, while the doctors are waiting for approval from the family to donate organs, many lives are lost.

Another respondent explained it as follows:

When someone has a donor card, no one needs to ask more than which organs correspond and to take those gifts for another person and save their life… life is important.

### The Priorities When Deciding About Organ Donation

The participants stated that they agree to donate and give authority when it is needed. However, the informants in this study emphasized that they have priorities in the event that their bodies are needed for donations. They mention several priorities in OD, from members of their family, through friends and acquaintances, to others. Regardless of the level of priority, all the respondents in the study indicated that they are willing to donate more than one organ to people when their authority is required.

#### Donation of Organs to Family Members

In the discussion about priorities in OD, all the subjects of this study stated that the first priority in organ donation would be in the context of family members. The subjects in this study were very well informed and had a great deal of knowledge about OD. All the participants in this study emphasized that, if they needed to donate an organ to someone from their family or someone not in their family, they would give priority to a donation to a family member in the majority of cases. Then, if it was possible, they would donate organs outside their family to other priority groups.

One respondent noted:

I’d donate organs to anyone, but, if my organs were needed by someone in my family, that person would have priority.

Another person said the following:

Organ donation first within the family and then for others, blood is thicker than water.

#### Donation of Organs to Friends

The participants agreed that they would also donate their organs to their friends. Friends of the respondents did not have to fulfill any extra conditions; they were the number two priority. Before them, the participants placed the members of their families as the number one priority.

One respondent said on this point:

I have many real friends and I would do everything for them. When it comes to organ donation, only in that case would members of my family be in front of my friends.

#### Organ Donation to Acquaintances and Others

The informants stated once again that they were willing to donate all their organs. As the last priority in OD, all the respondents agreed that their last priority would be acquaintances and others.

In a somewhat ridiculous sentence, one of the respondents said:

If my organs were needed for several people at one time, then let the best ones be taken for my family, the slightly less important organs for friends and acquaintances and the others can take what’s left.

### The Religious Aspects of Organ Donation

In the third main area, *the religious aspects of organ donation*, the participants in the present study focused on their view of OD from a religious perspective. The participants in the study agreed that all religions support and accept OD. The participants explained and underlined the importance of religion and religious affiliation for OD. They also explained the importance of the fact that religion must be studied and taught daily. They also pointed out that most people say that they are religious, although they have only learned a little about the religion to which they belong and know little about their religion. The participants in the study agreed that this could be the most important reason why members of different religions refuse OD.

#### The Attitude of Religion to Donating Organs

The participants in this study were well versed in their religion and knew exactly what their religious law (Fatwa) and all the points of that law say about OD. They also emphasized that it is important to know what their religion says about the issue of OD. The informants also stated the importance of removing fear and prejudice about OD.

One respondent said:

You cannot compare a person’s religion, when they have taught themselves or not, with a young educated person to whom information is available in any place and at any time.

A young respondent pointed out:

I often discuss religion and organ donation with my grandmother. She was born many years ago and heard about religion and organ donation from her parents.… She has her own opinion… I try to explain, but it’s no good.

#### Religious Education

Regarding religion and religious education, the participants in the study agreed that this is a key issue and a key question for a decision on OD. They stated that there are people who were “born” into their religion and practice religion in that way. On the other hand, there are people who were both born and have been educated in their religion and every day they perfect their religion and practice their religion in this field. Like everything else in the world, even religion must be constantly renewed and studied, according to all the participants in the study. However, they also noted that it is very difficult to change someone who was “born” into a religion and to educate and explain the religion in any way to those persons. All the participants agreed that this is one of the most important reasons why most religious people do not agree with OD.

One young participant said:

I often find myself in the company of older people and we discuss organ donation. They talk about things that neither religion nor science state and which are simply untrue.

When I try to explain to these people that their beliefs have nothing to do with religion, they get angry and say that I am still young and do not know enough about religion… What can I say?

## Discussion

The results of the present study demonstrate several characteristics of the informants. In general, the factors that might influence OD were access to information and knowledge about OD, priorities in OD, religious factors and religious beliefs. This was already shoved in previous studies (Tarhan et al. 2015; Krupic et al. 2017). However, in the present study, it was unexpected that the age, gender and religion of the informants in the present study did not influence their decisions regarding OD. All the informants agreed to donate their organs, regardless of age, gender or religion. It is important to note that the majority of the participants wanted to have information about organ donation, they were very motivated in relation to organ donation and they had looked for information in various ways. They searched for information on the Internet, TV, radio and other media, as well as from different healthcare institutions. The first reaction in communication with the participants highlighted the fact that they were very well informed about OD. The present study found that the participants emphasized the importance of adequate, correct information and the importance of holding donor cards. Information about OD was accessible and sufficient, which made them much more likely to accept potential organ donation. This is in opposition to one of our previous studies where we showed that some of the influencing factors concerning organ donation were mainly related to limited information from society as well as limited information from healthcare professionals (Krupic et al. 2017). The reason for this can be attributed to the fact that the informants are well educated, they come from different countries and are well integrated into Swedish society. All the informants speak more than three languages, all of them are employed and meet different people every day, all of them use the Internet and all of them are educated in their religion. All the informants in the present study agreed that information about OD is the basis for any potential OD procedure. Mass media, the Internet, TV, radio and other forms of communication are an essential source of information on different health issues and communication plays an essential role in the promotion of health, as well as forming the image of the health services. In previous studies, TV, radio, the Internet and the press were the most relevant and important means of communication and they have an unbeatable advantage in terms of their reputation and the number of users, which is increasing daily (Ríos et al. 2010a, b). It even seems reasonable to believe that specific means of communication may be used selectively to reach certain population subgroups, such as the informants in the present study, from Bosnia and Herzegovina, Macedonia, Kosovo and Croatia. If the perception of and information about transplantation are good, their predisposition toward OD will be positive (Martinez-Alarcon et al. 2011). This is very important in OD because the population of potential donors plays a fundamental role in OD (Zambudio et al. 2006; Rios et al. 2010a, b). This is true if the informants are well informed and have a good knowledge of OD, which was the case in the present study. Other previous studies conducted worldwide have indicated the significant relationship between knowledge, information and attitudes to OD (Alvaro et al. 2008; Bratton et al. 2011; Horton and Horton 1991). In terms of the influence on and predictors of the willingness to become a potential donor, the results of the present study indicate that all the informants in the present study were informed and they knew that all the major religions agree with OD. These findings in the present study are not in agreement with another study which reported a negative relationship between the belief that the integrity of the body should be maintained after death and the willingness to donate (Skowronski 1997), because participants in that study were not only religious, they were also highly educated in general and educated in their religion. All the participants in the present study made their decision regarding potential OD based on personal or religious beliefs. The participants described their decision to donate organs to other persons, but despite the fact that they were religious and well educated in their religion, they still had a hierarchy and priorities in the donation process, starting with family members, followed by friends and then others. The findings in the present study are in opposition to those in other studies and these results were unexpected, because other studies have shown that religious individuals are often non-donors and have negative attitudes to OD (Barcellos et al. 2005; Irving et al. 2012). The saying “blood is thicker than water” was also mentioned in the study, which was unexpected, but the participants in this study emphasized that they owned their bodies and that their bodies belonged to them, they have the right to do what they think is best with their bodies at the time.

In terms of the influence and predictors of the religious aspects of becoming a potential donor, the results in the present study indicate that all the informants, despite their age, gender or religion, agreed that they would be organ donors. All the participants in the study explained and underlined the importance of religion and religious affiliation for OD. They explained that it is important that religion is studied and taught daily. They also pointed out that most people call themselves religious, although they have learned very little about the religion to which they belong and know little about their religion. In one of the previous studies including 499 teachers from Bosnia and Herzegovina who belonged to the three biggest religions, Islam, Catholicism and Orthodox, most informants replied that they support the idea of donating organs both during life and after death. Regarding this question, there is a significant difference between the groups (*p *= 0.0063). To the question whether they are prepared to donate an organ of a deceased family member, most replied that they would consent to donating an organ, while a significant number also replied that they were not sure. The results show that there is no significant difference between the replies given by the groups (*p *= 0.7694). To the question regarding whom they were prepared to donate an organ, most said they were prepared to donate one to a member of their family, then to a close relative, while the least would donate to a stranger. The results show that there is a significant difference between the groups (*p *= 0.0002) (Sadic et al. 2016). The informants say that the knowledge they have was inherited from their parents and that they did not receive a religious education. This may explain some of the differences and difficulties that persons who are not educated in religion have, leading them to refuse organ donation. Perhaps the informants in previous studies were only “normal” religious persons rather than people who have received a religious education. All the informants in this study are highly educated, have received a religious education, are employed and identify themselves as religious and educated Muslims. Their support for OD was expected because previous studies, which have studied different factors that are certainly associated with positive attitudes to organ donation, included educational level, socio-economic status and younger age (Ashraf et al. 2005; Mossialos et al. 2008; Krupic et al. 2017; Sadic et al. 2016). In an other study the positive attitude to organ donation was associated with religious score (*p* = 0·015), marital status (*p *= 0·031), and knowledge score (*p *= 0·003). A high level of knowledge was associated with employment and the perception of organ donation as permitted in religion, whereas a positive attitude was associated with single marital status and high level of knowledge (Abukhaizaran et al. 2018).

In the present study, the general education the informants possess and their personal education in religion are combined. In this case, the result is very obvious and inevitable, because all the participants in this study identify themselves as potential organ donors to others in case of need. Perhaps this combination and the development of knowledge are precisely the key to more frequent and successful OD, providing assistance to people individually and to society and the healthcare system as a whole.

### Limitations

To the best of the author’s knowledge, this is the first study of its kind. However, the present study has some limitations. One may be that the interviews were held with other people present, which may have made the participants nervous, possibly making it difficult to concentrate on the interview and discussion. The lack of common background among participants may also have contributed to a reduction in participant openness, which is an important requirement for focus group interviews, although this was not evident. Another limitation may be that the investigator in the present study belongs to the same ethnic group as the participants, which may be regarded as a risk factor for impartiality in the planning, execution and analysis of the research, because of pre-conceptions.

## Conclusion

The decision to agree to OD is a complex issue, based strongly on personal beliefs. In the present study, it was shown that these factors did not influence the informants’ decision to agree to OD. Good communication through the media, good information about OD and a high educational level, as well as education in their religion, were predictors of the informants agreeing to OD. In view of the findings, and because religious officials, age and gender play an important role in society, the informants should be informed and educated in terms of their religion through various training courses on OD. High levels of education through religious education and good information via various media, as well as a good knowledge of the Swedish language, are predictors of better OD. In order to overcome religious ideology as a barrier to OD, and to promote increased OD in the future, specific intervention studies and the improved involvement of religious communities and education in schools and the healthcare system are vital and must be a starting point for improved OD.

## References

[CR1] Abukhaizaran N, Hashem M, Hroub O, Belkebir S, Demyati K (2018). Knowledge, attitudes, and practices of Palestinian people relating to organ donation in 2016: A cross-sectional study. Lancet.

[CR2] Alvaro EM, Siegel JT, Turcotte D, Lisha N, Crano WD (2008). Dominick A. Living kidney donation among Hispanics: A qualitative examination of barriers and opportunities. Progress in Transplantation.

[CR3] Ashraf O, Ali S, Ali SA, Ali H, Alam M, Ali A, Ali TM (2005). Attitude toward organ donation: A survey in Pakistan. Artificial Organs.

[CR4] Barcellos FC, Araujo CL, da Costa JD (2005). Organ donation: A population based study. Clinical Transplantation.

[CR5] Bratton C, Chavin K, Baliga P (2011). Racial disparities in organ donation and why. Current Opinion in Organ Transplantation.

[CR6] Bruzzone P (2008). Religious aspects of organ transplantation. Transplantation Proceedings.

[CR7] Chen JX, Zhang TM, Lim FL, Wu HC, Lei TF, Yeong PK, Xia SJ (2006). Current knowledge and attitudes about organ donation and transplantation among Chinese university students. Transplantation Proceedings.

[CR8] Conesa C, Ríos A, Ramírez P, Cantéras M, Rodríguez MM, Parrilla P (2006). Attitudes toward organ donation in rural areas of southeastern Spain. Transplantation Proceedings.

[CR9] Conesa C, Ríos A, Ramírez P, Rodríguez MM, Rivas P, Canteras M, Parrilla P (2003). Psychosocial profile in favor of organ donation. Transplantation Proceedings.

[CR10] Council of Europe. (2012). International figures on organ donation and transplantation. Transplant newsletter.

[CR11] Davis C, Randahawa G (2004). “Don’t know enough about it”—Awareness and attitudes towards organ donation and transplantation among black Caribbean and black African population in Lambeth, Southwark and Lewisham, UK. Transplantation.

[CR12] Duzenly Y (2005). Organ transplantation according to written Islamic sources. Journal of Medical Ethics-Law and History.

[CR13] Graneheim UH, Lundman B (2004). Qualitative content analysis in nursing research: Concepts, procedures and measures to achieve trustworthiness. Nurse Education Today.

[CR14] Horton RL, Horton PJ (1991). A model of willingness to become a potential organ donor. Social Science and Medicine.

[CR15] Irving MJ, Tong A, Jan S, Cass A, Rose J, Chadban S, Allen RD, Craig JC, Wong G, Howard K (2012). Factors that influence the decision to be an organ donor: A systematic review of the qualitative literature. Nephrology, Dialysis, Transplantation.

[CR16] Krupic F, Sayed-Noor AS, Fatahi N (2017). The impact of knowledge and religion on organ donation as seen by immigrants in Sweden. Scandinavian Journal of Caring Sciences.

[CR17] Kurz RS, Scharff DP, Terry T, Alexander S, Waterman A (2007). Factors influencing organ donation decisions by African Americans: A review of the literature. Medical Care Research and Review.

[CR18] Kvale, S. (1997). Den kvalitativa forskningsintervjun. (The qualitative research interview). In Swedish. Studentliteratur, Lund, Sweden.

[CR19] Martínez-Alarcón L, Ríos A, Sánchez J, Guzmán D, López-Navas A, Ramis G, Febrero B, Ramírez P, Parrilla P (2011). Do future journalists have a favorable attitude toward deceased donation?. Transplantation Proceedings.

[CR20] McLafferty I (2004). Focus group interviews as a data collecting strategy. Journal of Advanced Nursing.

[CR21] Miles MS, Frauman AC (1998). Public attitudes toward organ donation. Dialysis Transplant.

[CR22] Morgan M, Hooper R, Jones R (2006). Attitudes to kidney donation and registering as a donor among ethnic groups in the UK. Journal of Public Health.

[CR23] Mossialos E, Costa-Font J, Rudisill C (2008). Does organ donation legislation affect individuals’ willingness to donate their own or their relative’s organs? Evidence from European Union survey data. BMC Health Services Research.

[CR24] Park K, Moon JL, Kim SI, Kim YS (1999). Exchange-donor program in kidney transplantation. Transplantation Proceedings.

[CR25] Ríos A, Febrero B, López-Navas A, Martínez-Alarcón L, Sánchez J, Guzmán D, Ramírez P, Parrilla P (2010). From where do our children receive information about organ donation and transplantation?. Transplantation Proceedings.

[CR26] Ríos A, Martínez-Alarcón L, Sánchez J, Jarvis N, Parrilla P, Ramírez P (2010). German citizens in southeastern Spain: A study of attitude toward organ donation. Clinical Transplantation.

[CR27] Rumsey S, Hurford DP, Cole AK (2003). Influence of knowledge and religiousness on attitudes toward organ donation. Transplantation Proceedings.

[CR28] Sadic S, Sadic J, Krupic R, Fatahi N, Krupic F (2016). The influence of information and religion on organ donation, as seen by school teachers in Bosnia and Herzegovina. Materia Socio-Medica.

[CR29] Sharif A (2012). Organ donation and Islam-challenges and opportunities. Transplantation.

[CR30] Siminoff LA, Burant CJ, Ibrahim SA (2006). Racial disparities in preferences and perceptions regarding organ donation. Journal of General Internal Medicine.

[CR31] Skowronski J (1997). On the psychology of organ donation: Attitudinal and situational factors related to the willingness to be an organ donor. Basic and Applied Social Psychology.

[CR32] Swedish Health Care Act. (2015). The Act concerning the ethical review of research involving humans. http://www.epn.se. Accessed 31 Dec 2014.

[CR33] Tarhan M, Dalar L, Yildirimoglu H, Sayar A, Altin S (2015). The view of religious officials on organ donation and transplantation in the Zeytinburnu District of Istanbul. Journal of Religion and Health.

[CR34] The Swedish Health and Medical Services Act 1982:763. (2014). http://www.socialstyrelsen.se/regelverk. Accessed 15 June 2013.

[CR35] The World Medical Association Declaration of Helsinki. (1964). *Code of ethics* 1964. World Medical Association, Edinburgh.

[CR36] Wakefield CE, Watts KJ, Homewood J, Meiser B, Siminoff LA (2010). Attitudes toward organ donation and donor behavior: A review of the international literature. Progress in Transplantation.

[CR37] Wang L, Chang PC, Shih FJ, Sun CC, Jeng C (2006). Self-care behavior, hope and social support in Taiwanese patients awaiting heart transplantation. Journal of Psychosomatic Research.

[CR38] Wilow K (2013). Författningshandbok.

[CR39] Zambudio AR, Conesa C, Ramírez P, Galindo PJ, Martínez L, Rodríguez MM, Parrilla P (2006). What is the attitude of hospital transplant-related personnel toward donation?. Journal of Heart and Lung Transplantation.

